# NFATc3 Promotes Pulmonary Inflammation and Fibrosis by Regulating Production of CCL2 and CXCL2 in Macrophages

**DOI:** 10.14336/AD.2022.1202

**Published:** 2023-08-01

**Authors:** Yunjuan Nie, Xiaorun Zhai, Jiao Li, Aijuan Sun, Huilian Che, John W Christman, Gaoshang Chai, Peng Zhao, Manjula Karpurapu

**Affiliations:** ^1^Department of Basic Medicine, Wuxi School of Medicine, Jiangnan University, Wuxi 214122, China.; ^2^Department of Pathology, Wuxi People's Hospital Affiliated to Nanjing Medical University, Wuxi 214023, China.; ^3^Pulmonary, Critical Care and Sleep Medicine, Davis Heart and Lung Research Institute, Ohio State University Wexner Medical Center, Columbus, Ohio, USA.

**Keywords:** Idiopathic pulmonary fibrosis, NFATc3, macrophage, CCL2, CXCL2

## Abstract

Idiopathic pulmonary fibrosis (IPF) is a progressive and highly lethal inflammatory interstitial lung disease characterized by aberrant extracellular matrix deposition. Macrophage activation by cytokines released from repetitively injured alveolar epithelial cells regulates the inflammatory response, tissue remodeling, and fibrosis throughout various phases of IPF. Our previous studies demonstrate that nuclear factor of activated T cells cytoplasmic member 3 (NFATc3) regulates a wide array of macrophage genes during acute lung injury pathogenesis. However, the role of NFATc3 in IPF pathophysiology has not been previously reported. In the current study, we demonstrate that expression of NFATc3 is elevated in lung tissues and pulmonary macrophages in mice subjected to bleomycin (BLM)-induced pulmonary fibrosis and IPF patients. Remarkably, NFATc3 deficiency (NFATc3^+/-^) was protective in bleomycin (BLM)-induced lung injury and fibrosis. Adoptive transfer of NFATc3^+/+^ macrophages to NFATc3^+/-^ mice restored susceptibility to BLM-induced pulmonary fibrosis. Furthermore, in vitro treatment with IL-33 or conditioned medium from BLM-treated epithelial cells increased production of CCL2 and CXCL2 in macrophages from NFATc3^+/+^ but not NFATc3^+/-^ mice. CXCL2 promoter-pGL3 Luciferase reporter vector showed accentuated reporter activity when co-transfected with the NFATc3 expression vector. More importantly, exogenous administration of recombinant CXCL2 into NFATc3^+/-^ mice increased fibrotic markers and exacerbated IPF phenotype in BLM treated mice. Collectively, our data demonstrate, for the first time, that NFATc3 regulates pulmonary fibrosis by regulating CCL2 and CXCL2 gene expression in macrophages.

## INTRODUCTION

IPF is a progressive interstitial lung disease characterized by a profibrotic wound-healing cascade, chronic inflammation and accumulation of fibroblasts, ultimately leading to respiratory failure and death [1]. Despite extensive research, the etiology and mechanisms of IPF have not been fully established. As a result, current therapeutic approaches for IPF are limited, resulting in a median postdiagnosis survival of only 2 to 3 years [2]. Aberrant wound healing and remodeling of type II alveolar epithelial cells (AECs) are important drivers of IPF [3, 4]. Furthermore, macrophages are the most abundant immune cells in the lungs, which can exhibit either pro-inflammatory or pro-fibrotic phenotypes and play a regulatory function in wound healing responses [5]. Upon activation by cytokines such as IL-33 which is released from injured AECs, macrophages can produce multiple pro-inflammatory cytokines and chemokines such as tumor necrosis factor α (TNF-α), IL-1β, C-C motif ligand 2 (CCL2) and CCL12, which initiate inflammation and induce myofibroblast activation, thereby contributing to lung fibrosis [6, 7]. Therefore, understanding molecular mechanisms of macrophage phenotypic activation is essential to target IPF pathophysiology effectively.

Initially discovered in T-cells, the Nuclear Factors of Activated T-cell (NFAT) family proteins are transcription factors studied extensively for their diverse regulatory roles in cellular development, immune cell function and inflammatory responses in diverse cell types [8-10]. Of the five proteins in the NFAT family, NFAT1, NFAT2, NFAT3, and NFAT4 are regulated by cellular calcium influx, whereas NFAT5 is regulated by osmotic stress [11, 12]. NFATs directly bind to the target gene promoters or form cooperative complexes with other transcription factors (NF-κB and AP-1) to regulate the transcription of multiple cytokine genes. NFATs have been implicated in a range of inflammatory diseases such as cholestasis, skin edema, and acute lung injury by regulating the macrophage inflammatory phenotype [13-15]. To our knowledge, the role and regulatory mechanism of NFAT proteins in the pathogenesis of pulmonary fibrosis have not been reported.

In the current study, we demonstrate that NFATc3 is the only member of NFATs elevated in the lung tissues and pulmonary macrophages of IPF patients and mice. Interestingly, NFATc3^+/+^ mice subjected to BLM-induced pulmonary fibrosis showed increased accumulation of fibrotic foci, extracellular matrix protein deposition, fibronectin, α-SMA, CCL2 and CXCL2 production. In contrast, all the fibrotic markers, Trichrome blue staining and Ashcroft score indicating fibrosis severity were significantly attenuated in NFATc3+/- mice subjected to BLM-induced pulmonary fibrosis. NFATc3^+/-^ mice that received NFATC3^+/+^ pulmonary macrophages by adoptive transfer or recombinant mouse CXCL2 alone showed increased pulmonary fibrosis severity and fibrotic gene expression similar to wild-type (NFATC3^+/+^) mice, suggesting a pivotal role for NFATc3 in the development of lung fibrosis. Based on these observations, we sought to determine the molecular mechanisms by which macrophage NFATc3 regulates pulmonary fibrosis using mouse BLM-induced IPF models.

## MATERIALS AND METHODS

### Mice

NFATc3^+/-^ mice purchased from the Beijing Viewsolid Biotech Co. LTD (Beijing, China) were backcrossed onto C57BL/6 mice for around 11 generations. C57BL/6 mice were purchased from Slac Laboratory Animal Co., Ltd. (Shanghai, China). Mice were housed in specific pathogen-free conditions at the Laboratory Animal Center of JiangNan University. All the procedures involving mice were approved by the Institutional Animal Care and Use Committee at JiangNan University (JN. No 20211130m1720615[501]).

### BLM-induced pulmonary fibrosis

After anesthesia, 1.4 U/kg of BLM (Cat#RB003, BioTang, USA) in 50 μL of sterile saline was delivered into wild-type (WT, referred as NFATc3^+/+^) and NFATc3^+/-^ mice (8-10 weeks old) via an intratracheal route. Mice administered with the same volume of saline served as controls. The mice were euthanized 3, 7 or 21 days after BLM or saline challenge for the analysis of pulmonary inflammation and fibrosis.

### Bronchoalveolar lavage fluid (BALF) collection and analysis

BALF was collected from IPF mice on day 3 and 7 by cannulating the trachea and lavaging the lung with 1 ml of sterile PBS. BALF was centrifuged at 4^o^C, 200g for 15 minutes. Total cells in BALF pellet were resuspended in PBS and counted. Cell free BALF supernatant was analyzed for extravasated protein [16].

### Lung histology

The left lung was inflated with 4% paraformaldehyde via an intratracheal route, then removed and placed in fresh 4% neutral buffered paraformaldehyde for 48 hours, followed by dehydration, paraffin embedding and histological analysis for Hematoxylin and Eosin (H&E) or Masson trichrome staining (D026-1-3, NanJing Jiancheng Bioengineering Institute, China) as previously described [17]. The degree of pulmonary fibrosis was evaluated using the modified Ashcroft score, where fibrosis severity is scored from 0 to 8 [18].

### Immunohistochemistry

For immunohistochemical staining, left lung sections from murine IPF models and IPF patients (around 2CM*2CM) were incubated at 4°C overnight with anti-NFATc3 antibody (4998, Cell Signaling, USA). In parallel, lung sections incubated with diluent only were included as controls to compare false positive staining. Immunoreactive antigens were detected using Avidin-Biotin Complex and visualized with diaminobenzidine (AR1022, BOSTER Biological Technology, China). Lung sections from IPF patients were kindly provided by Wuxi People’s Hospital (Jiangsu, China), using procedures approved by the Institutional Review Board of Wuxi People’s Hospital (JNU20220310IRB31).

### Cell culture

MLE12 cells were cultured in HITES medium (Ham's F12, 50:50) containing 10% fetal bovine serum (0500, Gibco, USA), 100 U/ml penicillin, and 100 μg/ml streptomycin. Bone marrow-derived macrophages (BMDMs). Bone marrow cells were collected from femurs and tibia of NFATc3^+/+^ and NFATc3^+/-^ mice and cultured in Dulbecco’s modified eagle medium (DMEM) containing 10% FBS, 1% penicillin/streptomycin, and 10% L929 medium for 7 days. The culture medium was replaced with fresh DMEM containing L929, FBS and penicillin/streptomycin after 3 and 5 days and differentiated BMDMs after 7 days of culture were used in cellular assays.

Alveolar macrophages (AMs). BALF from control and BLM-treated mice was centrifuged for 15 min at 200 g and 4°C. Cell pellet was treated with 1X RBC lysis buffer (C3702, Beyotime Biotechnology, China), incubated on tissue culture plates for 1hour, washed with Ca+2 and Mg+2 free PBS to collect adhering AMs.

Interstitial macrophages (IMs). IMs were isolated from the whole lungs of mice via collagenase digestion as described previously (24). Briefly, lungs were minced with scissors and incubated at 37°C for 40 minutes in digestion buffer containing RPMI, 10% fetal calf serum, 1 mg/ml collagenase (C5138-1G, Sigma, USA), 30 μg/ml DNase (D4263-1VL, Sigma-Aldrich, USA) per lung. Red blood cells from total lung cells were removed by incubating in RBC lysis buffer and rinsing with PBS. IMs from total lung cells were allowed to adhere to tissue culture dishes for 1 hour in serum free RPMI, that was replaced with complete RPMI for overnight incubation. IM purity was determined by flow cytometry (>95%) as described previously [17, 19].

### Adoptive transfer of macrophages

Adoptive transfer of mouse macrophages was performed as described previously [17, 20]. First the resident pulmonary macrophages of NFATc3^+/+^ and NFATc3^+/-^ mice were depleted by i.t delivery of liposomes encapsulating clodronate (50 μL). 2 days after clodronate liposomal delivery, NFATc3^+/+^ and NFATc3^+/-^ recipient mice were administered with BMDMs (i.t 5 ^10^5^ cells per mouse in 50 μl saline) of different NFATc3 background (NFATc3^+/-^ to NFATc3^+/+^ and NFATc3^+/+^ to NFATc3^+/-^). Mouse that received NFATc3^+/-^ to NFATc3^+/-^ or NFATc3^+/+^ to NFATc3^+/+^ BMDMs served as controls. After 24h of BMDM adoptive transfer, all the four groups of mice were subjected to BLM-induced IPF and analyzed.

### Isolation of total RNA and quantitative PCR

Total RNA from the lung tissues and macrophages were isolated using Trizol reagent (Life Technologies, USA) according to the manufacturer’s protocols. Isolated RNA was reverse transcribed into cDNA with a PrimeScript RT Reagent Kit (DRR019A, TaKaRa, Otsu, Shiga, Japan). Expression levels of individual genes was measured by quantitative PCR with SYBR Premix Ex Taq (11198ES03, Yeasen, China) on a LightCycler® 480 PCR detection system (AXYPCR96LC480WNF, Roche, Foster City, CA, USA). Quantification was performed using the 2(^-∆∆^Ct) method nomalized to GADPH. The gene-specific primers used are listed below: a-SMA (forward 5’-GACGCTGAAGTATCCGATAGAACAC G-3’, reverse 5’-CACCATCTCCAGAGTCCAGCACA AT-3’); fibronectin (forward 5’-TCTGGGAAATGGAA AAGGGGAATGG-3’, reverse 5’-CACTGAAGCAGGT TTCCTCGGTTGT-3’); TNF-α (forward, 5’-TTCTCAT TCCTGCTTGTGG-3′, reverse, 5’-ACTTGGTGGTTTG CTACG-3’); IL-1β (forward, 5′-CCAGCTTCAAATCTC ACAGCAG-3’; reverse, 5′-CTTCTTTGGGTATTGCTT GGGATC-3’); IL-12p35 (forward, 5′-GGACCAAACCA GCACAT-3′; reverse, 5′- CGCAGAGTCTCGCCATTA-3’); IL-12p40 (forward, 5′- TGAACTGGCGTTGGAAG-3’; reverse, 5’-GAAGTAGGAATGGGGAGTG-3’); CCL2 (forward 5′-TCTGGACCCATTCCTTCTTGG -3’,

reverse 5’-TCAGCCAGATGCAGTTAACGC -3’); CX CL2 (forward 5’-CCTGGTTCAGAAAATCATCCA -3’,

reverse 5’-CTTCCGTTGAGGGACAGC-3’); IL-13 (forward 5’-GAATCCAGGGCTACACAGAAC-3’, reverse 5’-AACATCACACAAGACCAGACTC-3’); TGF-β1 (forward 5′-TGTACGGCAGTGGCTGAAC CAA-3’, reverse 5’-CGCTGAATCGAAAGCCCTGTA TT-3’); and GAPDH (forward 5’-TGCGACTTCAAC AGCAACTC-3’, reverse 5’-CTTGCTCAGTGTCCTTG CTG-3’).

### Hydroxyproline Assay

Left lungs were hydrolyzed and total lung hydroxyproline levels were quantified using hydroxyproline assay kit (A030-2-1, NanJing Jiancheng Bioengineering Institute, China). Each sample was tested in triplicate. Data are expressed as micrograms of hydroxyproline per gram left lung.

### Dual-luciferase reporter assay

The putative NFAT binding sites on CXCL2 promoter (from -1000 bp to +200 bp) were predicted using the JASPAR database 6. The CXCL2 promoter encompassing NFAT consensus binding sequence was subcloned into the pGL3-basic luciferase reporter plasmid vector (VT1554, YouBio, China) and confirmed by DNA-sequencing using the BigDye fluorescent label termination substrate cycle sequencing kit on an automated sequencing Analyzer (model 3730, PEABI, USA).

The pGL3- CXCL2 promoter luciferase plasmid or control pGL3-luciferase plasmid was transiently co-transfected with NFATc3 expressing plasmid NFATc3-pDON223 or control plasmid-pDON223 into RAW264.7 cells using Lipo3000 transfection reagent (L3000150, ThermoFisher Scientific, USA). Renilla luciferase vector was used as an internal control for normalization. 48 h after transfection, the NFAT driven luciferase activity was measured using Dual Luciferase Reporter Assay Kit (11402ES60, Yeasen, China) according to manufacturer’s instructions and expressed as firefly luciferase activity normalized to Renilla luciferase activity.

**Figure 1.**
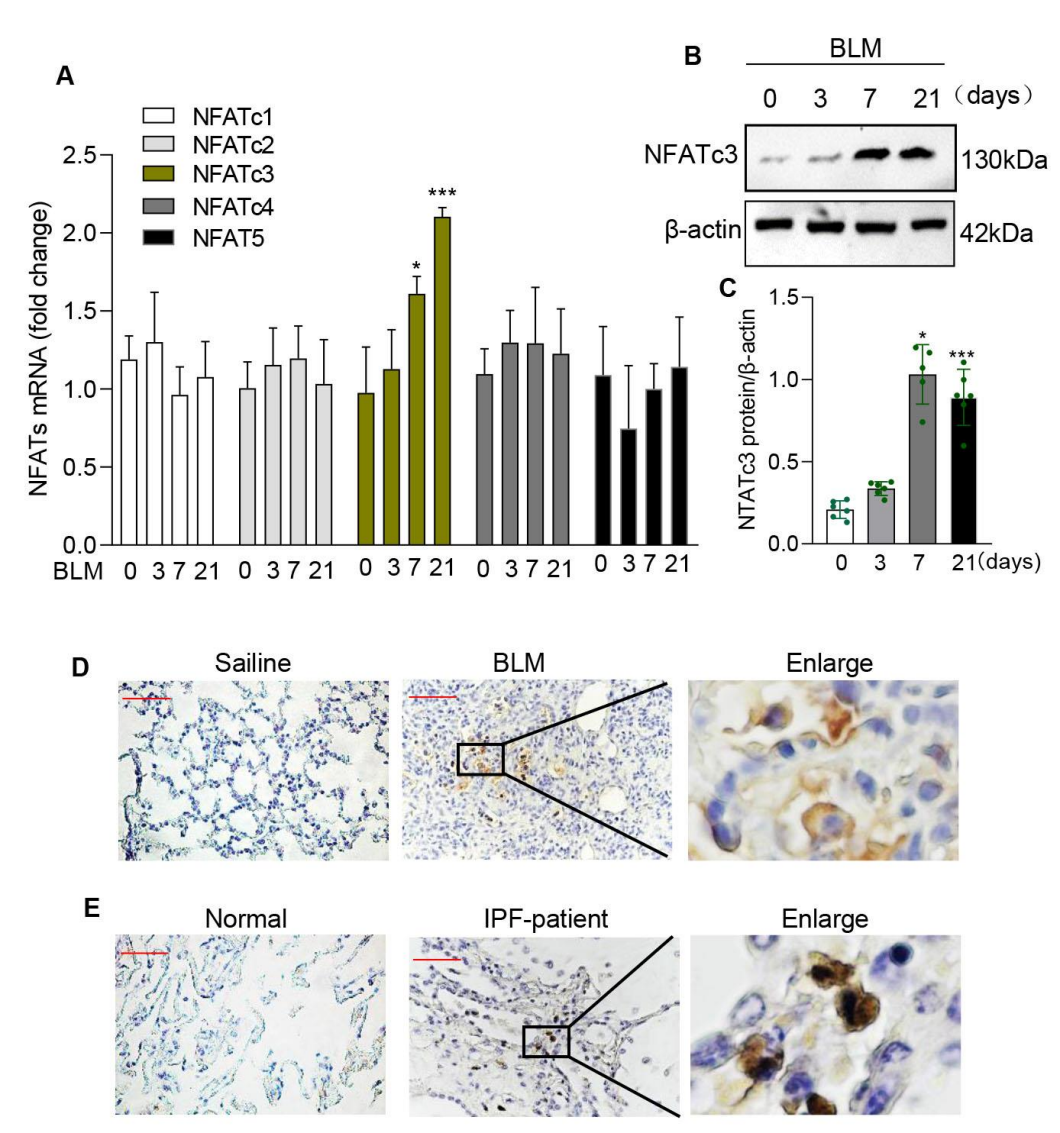
**NFATc3 expression is elevated in alveolar and interstitial macrophages of bleomycin treated IPF mice and IPF patients**. NFATc3^+/+^ (WT) mice were treated with BLM (1.4 U/kg, i.t) and lung tissue were analyzed on day 0, 3, 7 and 21. (**A**) The expression levels of NFATc1, NFATc2, NFATc3, NFATc4 and NFATc5 measured by real time RT-PCR. (**B**) The protein level of NFATc3 was analyzed by western blotting. (**C**) Arbitrary densitometry units of NFATc3 normalized to β-actin calculated using Image J software. (**D**) Immunohistochemical staining of NFATc3 in lung tissue from IPF or control mice (original magnification ×400, scale bar 100μm). (**E**) Immunohistochemical staining of NFATc3 in lung tissue from healthy donor or pulmonary fibrosis patients (original magnification ×400, scale bar 100μm). Data are shown as mean ± SEM. N =6 for each group, *p < 0.05, **p < 0.01, ***p < 0.001 (Saline vs 7, 21 days of BLM).

**Figure 2.**
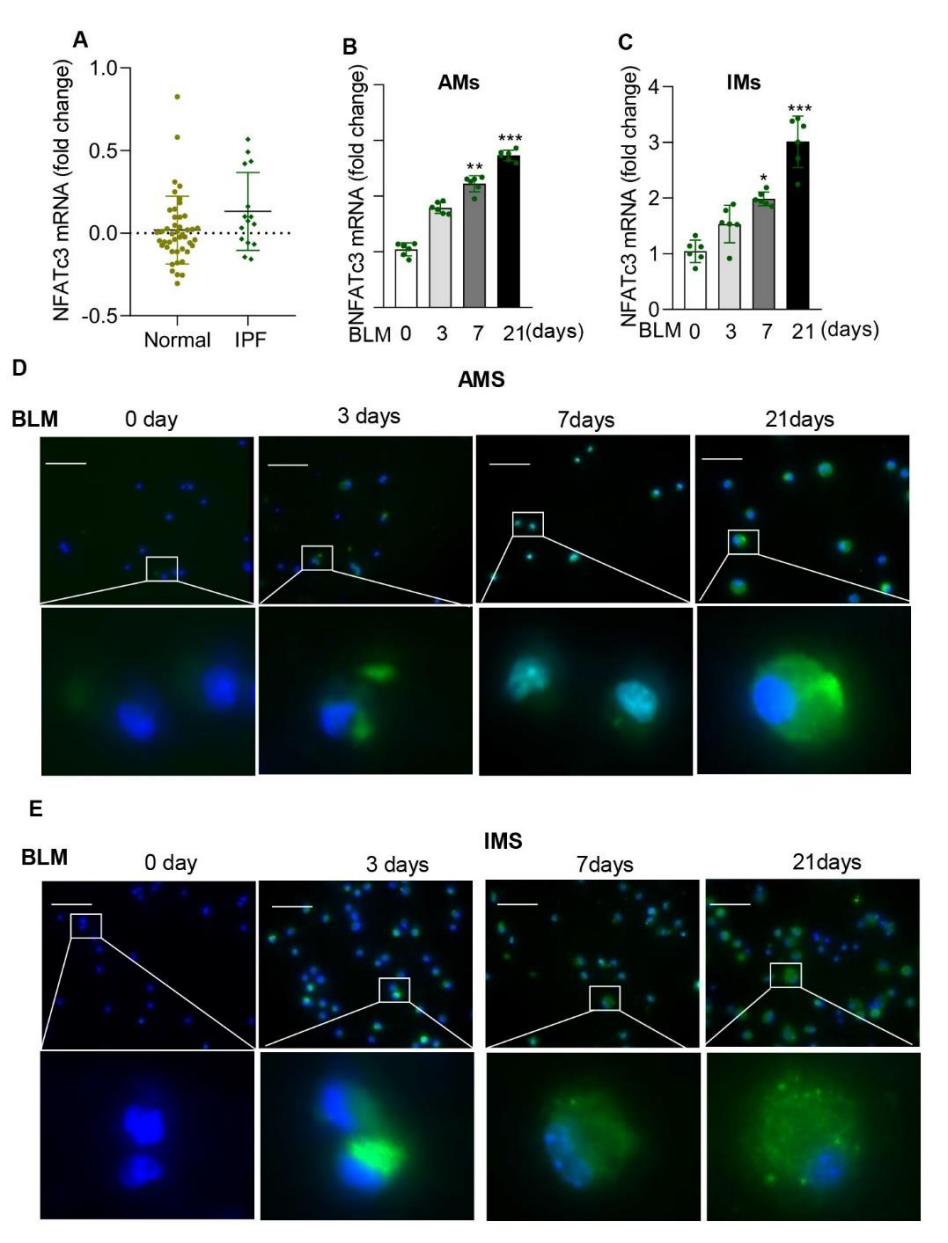
**NFATc3 expression is elevated in alveolar and interstitial macrophages of IPF patients and mice**. (**A**) NFATc3 gene expression by microarray analysis of alveolar macrophage (AM) mRNA in healthy control subjects (N=45) and sporadic IPF patients (N=15). NFATc3^+/+^ mice were treated with BLM (i.t) to induce pulmonary fibrosis, and after 3, 7 and 21 days, NFATc3 expression in (B) AMs and (C) IMs was detected by RT-qPCR analysis (N=6 for each group). (**D, E**) NFATc3 nuclear translocation in AMs and IMs was detected by immunofluorescence staining (scale bar 50μm). Data are shown as mean ± SEM. N =6 for each group, *p < 0.05, **p < 0.01, ***p < 0.001 (Saline vs 3, 7, 21 days of BLM).

### Western blotting

Protein expression was analyzed by Western blotting. In brief, total proteins from lung tissues were extracted with the 1X RIPA lysis buffer, resolved by sodium dodecyl sulfate polyacrylamide gel electrophoresis, transferred to a polyvinylidene difluoride membrane, and incubated with a primary antibody against NFATc3 and β-actin antibodies (3700, Cell Signaling Technology, Beverly, USA). After incubation with peroxidase-conjugated secondary antibodies, the signals were visualized by enhanced chemiluminescence (U10012, UUBIO, China) according to the manufacturer’s instructions. The band intensity was quantified using image J software (US National Institutes of Health, Bethesda, MD, USA).

### Statistical analysis

Data were expressed as mean±SEM. The *in vitro* data were obtained from at least three independent experiments, and *in vivo* data were obtained from experiments by at least 6 mice in each group. Statistical differences between single comparisons were performed by Student’s t-test, and the differences between two groups were performed by Mann-Whitney tests, multiple groups by non-parametric ANOVA Kruskall-Wallis test using GraphPad Prism 9. Statistical significance was defined as *p< 0.05, **p <0.01, ***p <0.001.

**Figure 3.**
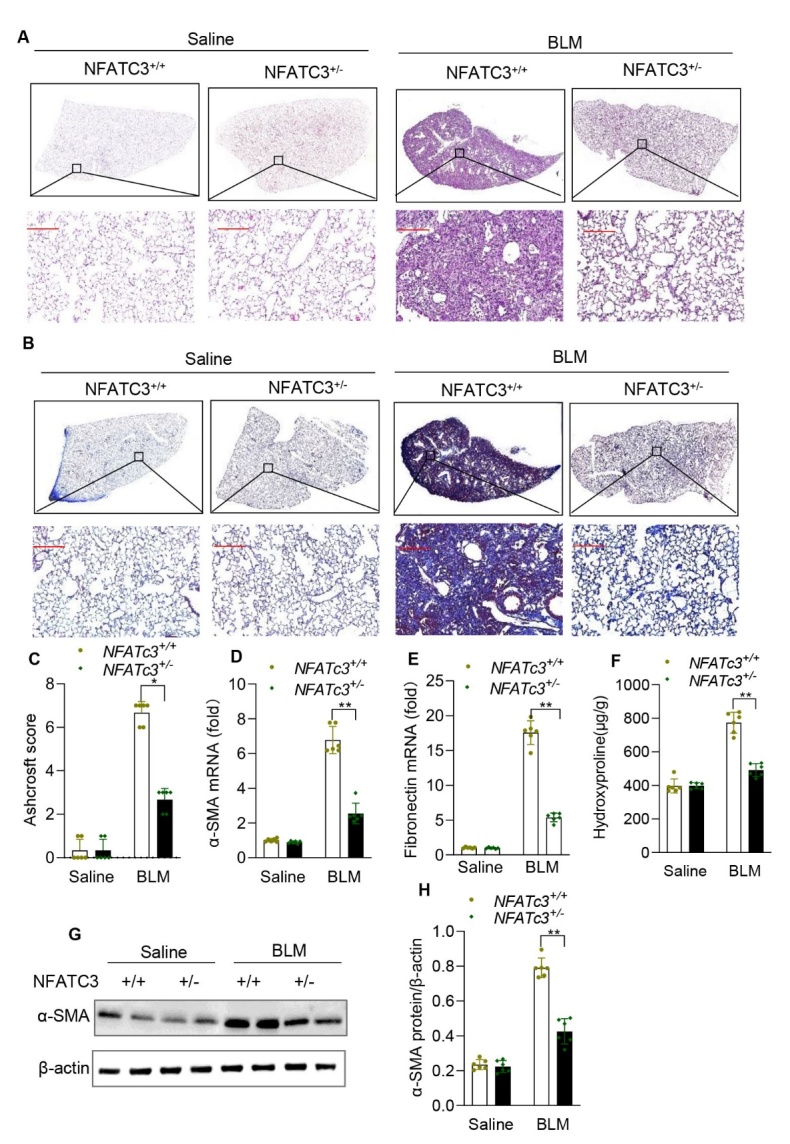
**NFATc3 deficiency alleviated bleomycin-induced pulmonary fibrosis in mice**. NFATc3^+/+^ and NFATc3^+/-^ mice were treated with bleomycin (1.4 U/kg, i.t) to establish pulmonary fibrosis. Sham control mice received the same volume of saline (i.t). After 21 days, lung tissue sections were stained by (A) hematoxylin-eosin (HE) and (B) Masson trichrome staining (original magnification ×400, scale bar100μm). (**C**) Severity of Pulmonary fibrosis among different experimental groups was compared by Ashcroft score. (**D, E**) Total RNA was extracted from lung tissues and the expression levels of ɑ-SMA and fibronectin were detected by RT-q-PCR. (**F**) Hydroxyproline was measured in different groups of mice. (**G, H**) The protein levels of ɑ-SMA were analyzed by western blotting and quantified using Image J software. Data are shown as mean ± SEM. N =6 for each group, *p < 0.05, **p < 0.01, ***p < 0.001 (NFATc3^+/+^ vs NFATc3^+/-^).

**Figure 4.**
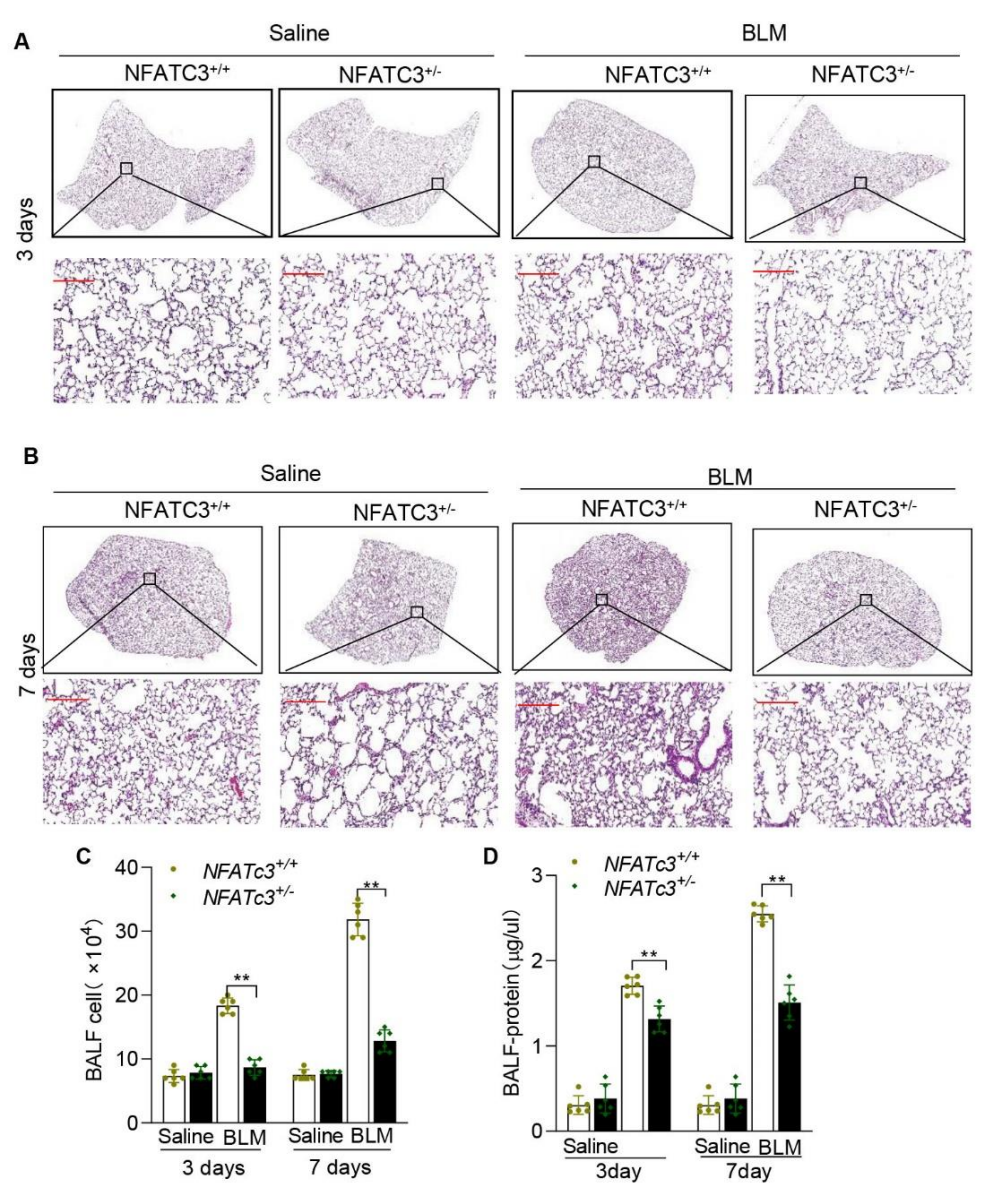
**NFATc3 deficiency decreased BLM-induced pulmonary inflammation in mice**. NFATc3^+/+^ and NFATc3^+/-^ mice were treated with normal saline or bleomycin (i.t) for 3 and 7 days. (**A, B**) lung tissue sections were stained by hematoxylin-eosin (HE) (scale bar 100μm). (**C**) Immune cell infiltration in to BALF was determined by counting total cells (D) Alveolar damage and protein leak in to BALF was determined by quantitating total BALF protein. Data are shown as mean ± SEM. N =6 for each group, *p < 0.05, **p < 0.01, ***p < 0.001 (NFATc3^+/+^ vs NFATc3^+/-^).

## RESULTS

### NFATc3 expression is elevated in IPF patients and BLM-induced mouse fibrosis models

Although pulmonary macrophages are known to be involved in the development of IPF, the distinct function of macrophage specific NFAT isoforms in pulmonary fibrosis remains unclear. Analysis of BLM-treated mouse lung tissue showed a significant increase in NFATc3 mRNA expression levels on days 7 and 21 compared to other NFATs (Fig. 1A). Similarly, higher levels of NFATc3 protein expression were observed in the lung tissues of BLM-induced IPF mice (Fig. 1B, C). Immunohistochemical staining of lungs from IPF mice and patients confirmed the upregulated expression of NFATc3 when compared to non-IPF control lungs and NFATc3-positive cells to be macrophages from their shape and morphology (Fig. 1D and E). To further determine whether the NFATc3 expression is elevated and activated in pulmonary macrophages of IPF patients, we compared NFATc3 expression level between 45 healthy control subjects and 15 subjects with sporadic IPF from the GEO profiles (GSE 49072), the result show significantly increased mRNA level of NFATc3 in pulmonary macrophages of sporadic IPF patients (Fig. 2A). However, the difference was not statistically significant, partly due to the smaller sample size of the IPF patients compared to the control group. Alveolar macrophages (AMs) and interstitial macrophages (IMs) were isolated from BLM-induced IPF mice for further analysis. The NFATc3 expression assessed by real time PCR displayed notably upregulated in both AMs and IMs with a time dependent manner on day 3, 7, and 21 (Fig. 2B and C). Moreover, NFATc3 nuclear localization was enhanced in these primary cells from IPF mouse models (Fig. 2D and E). Taken together, these data suggest that NFATc3 expression and cytoplasmic to nuclear translocation occurs in pulmonary macrophages correlate with the progress of pulmonary fibrosis development.

**Figure 5.**
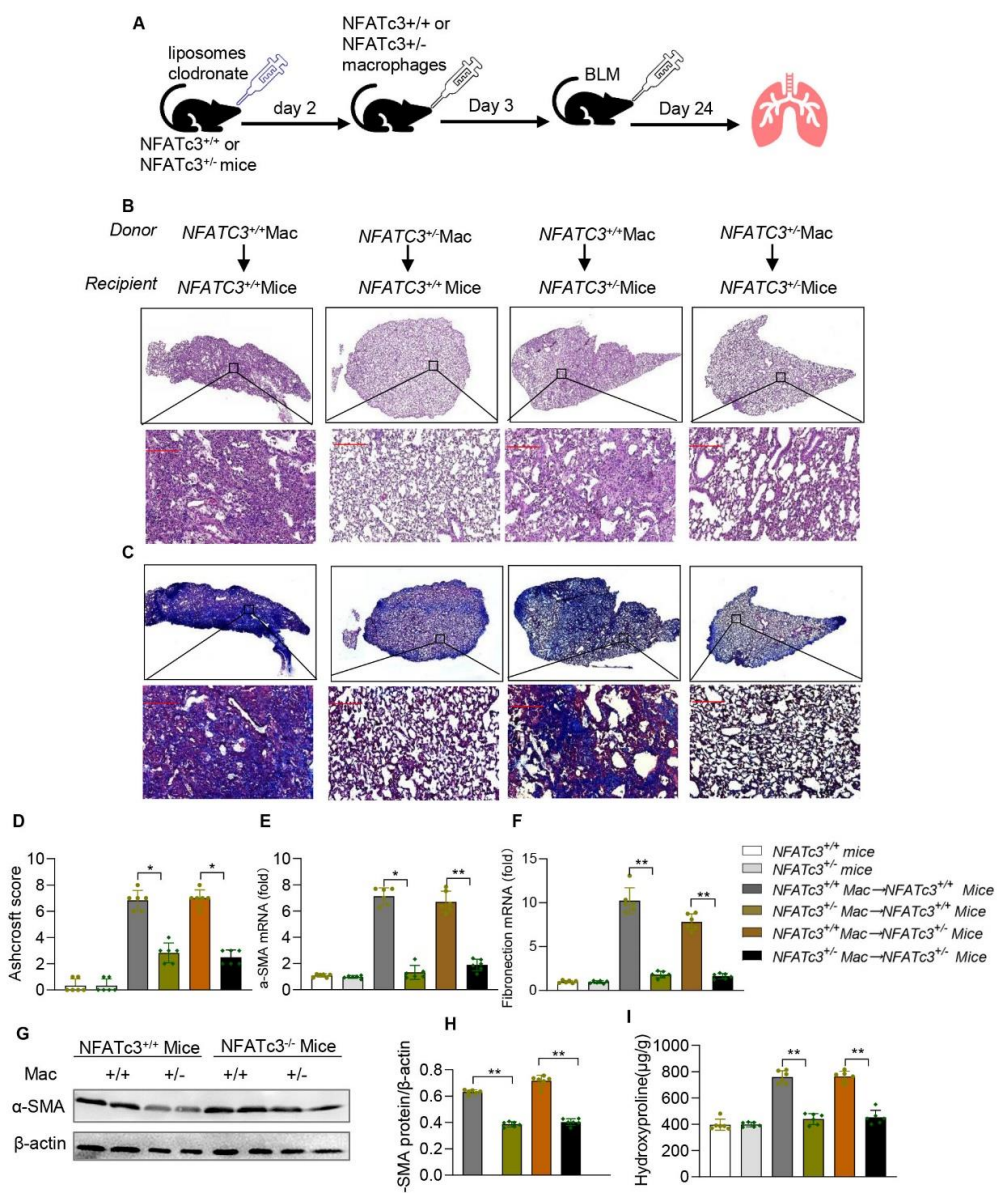
**NFATc3 expression in pulmonary macrophages enhances susceptibility to BLM-induced IPF in mice**. (**A**) Schematic diagram for the adoptive transfer of macrophages described in Materials and Methods. NFATc3^+/+^ or NFATc3^+/-^ mice were adoptively transferred with NFATc3^+/+^ or NFATc3^+/-^ BMDMs, and then treated with BLM (i.t) for 21 days. Lung sections were stained with (B) H&E to detect overall histological changes, and (C) Masson’s trichrome staining to detect collagen deposition (original magnification ×400, scale bar 100μm). (**D**) Severity of fibrosis was expressed as a means of individual Ashcroft scores of different experimental animals. (**E, F**) RNA was extracted from individual lung tissues and the mRNA levels of ɑ-SMA and fibronectin were detected by RT-qPCR. (**G, H**) The protein level of ɑ-SMA was analyzed by western blotting and quantified using Image J software. (**I**) Hydroxyproline was measured using hydroxyproline assay kit in different groups of mice. Data are shown as mean ± SEM. N =6 for each group, *p < 0.05, **p < 0.01, ***p < 0.001 (NFATc3^+/+^Macrophages→ NFATc3^+/+^/ NFATc3^+/-^ mice vs NFATc3^+/-^Macrophages→ NFATc3^+/+^/ NFATc3^+/-^ mice).

**Figure 6.**
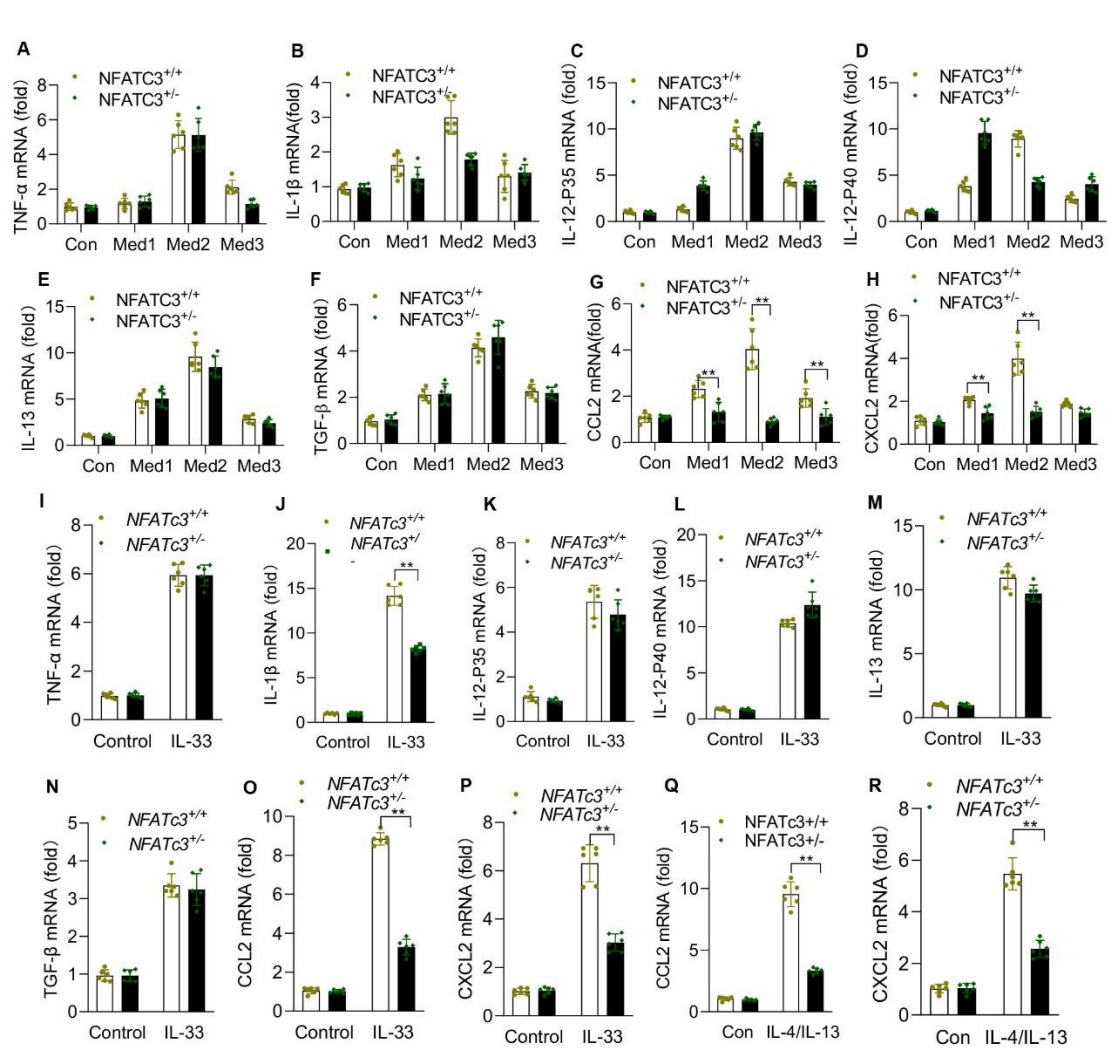
**NFATc3 regulates mRNA expression of CCL2 and CXCL2 in macrophages**. MLE12 cells were cultured and stimulated with different concentrations of bleomycin, 0.05 nM, 0.5 nM and 5 nM for 24 hours, respectively. NFATc3^+/+^ and NFATc3^+/-^ BMDMs were stimulated with different concentration of bleomycin-treated MLE12 culture supernatant (indicated as Med1, Med2 and Med3) for 24 hours, and then total RNA was extracted. The mRNA levels of (A) TNFα (B) IL-1β (C) IL-12P35 (D) IL-12P40 (E) IL-13 (F) TGF-β (G) CCL2 and (H) CXCL2 were measured by RT-qPCR. BMDMs from NFATc3^+/+^ and NFATc3^+/-^ mice were treated separately with IL-33 (10 ng/ml) for 24, and the mRNA levels of (I) TNFα (J) IL-1β (K) IL-12P35 (L) IL-12P40 (M) IL-13 (N) TGF-β (O) CCL2 and (P) CXCL2 were measured by RT-qPCR. (**Q, R**) BMDMs from NFATc3^+/+^ and NFATc3^+/-^ mice treated with Th2 cytokine mix (IL-4 and IL-13, 10 ng/mL each for 24 h) were analyzed for CCL2 and CXCL2 expression. Each experiment was independently repeated in triplicate, with duplicated wells. Data are shown as mean ± SEM. *p < 0.05, **p < 0.01, ***p < 0.001 (NFATc3^+/+^ vs NFATc3^+/-^).

### NFATc3 deficiency attenuates BLM-induced lung fibrosis

Based on the above observations, we next investigated the functional role of NFATc3 on pulmonary fibrosis development by using NFATc3^+/-^ and NFATc3^+/+^ mice, subjected to BLM treatment for 21 days. Histological analysis showed multifocal fibrotic pulmonary lesions, with accumulated fibroblasts, myofibroblasts, and extracellular matrix deposition in the lungs of WT mice after the BLM challenge (Fig. 3A). In contrast, the degree of pulmonary fibrosis is significantly lower in NFATc3^+/-^ mice (Fig. 3A). We further assessed the degree of pulmonary fibrosis using Masson’s trichrome staining and found that there was less collagen deposition (blue pigment) in the lung interstitium of NFATc3^+/-^ compared to NFATc3^+/+^ mice, indicating decreased severity of fibrosis (Fig. 3B). Furthermore, the pathological scores of fibrosis at 21 days after treatment with BLM was also less in NFATc3^+/-^ mice as compared to wild-type mice (Fig. 3C). Consistent with fibrosis scores, expression of the other fibrotic markers, α-smooth muscle cell actin (α-SMA) and fibronectin decreased significantly in NFATc3^+/-^ fibrosis mice (Fig. 3D and E). We also observed that α-SMA protein and hydroxyproline were decreased in BLM-treated NFATc3+/- compared to NFATc3+/+ mice (Fig. 3G-H and F, respectively). These data indicate that NFATc3 gene knockdown can significantly attenuate BLM-induced fibrosis markers.

**Figure 7.**
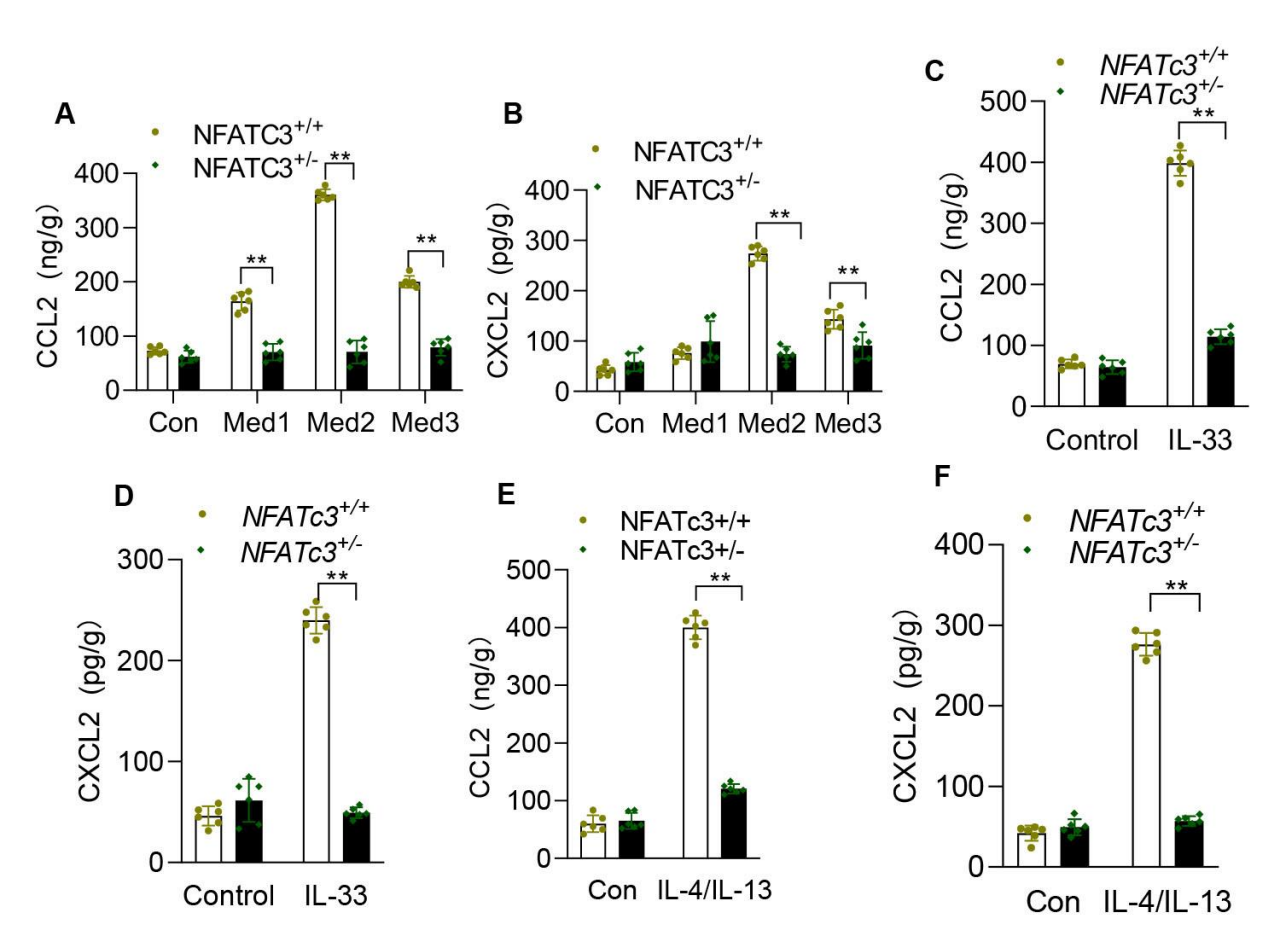
**NFATc3 regulates protein levels of CCL2 and CXCL2 in macrophages**. (**A, B**) NFATc3^+/+^ and NFATc3^+/-^ BMDMs were stimulated with different concentrations of bleomycin-treated MLE12 culture supernatant (indicated as Med1, Med2 and Med3) for 24 h, and the protein levels of CCL2 and CXCL2 were detected using ELISA. (**C, D**) BMDMs from NFATc3^+/+^ and NFATc3^+/-^ mice were treated separately with IL-33 (10 ng/ml) for 24, and the protein levels of CCL2 and CXCL2 were detected by ELISA. (**E, F**) BMDMs from NFATc3^+/+^ and NFATc3^+/-^ mice treated with Th2 cytokine mix (IL-4 and IL-13,10 ng/mL each for 24 h) were analyzed for CCL2 and CXCL2 expression. Each experiment was independently repeated in triplicate, with duplicated wells. Data are shown as mean ± SEM. *p < 0.05, **p < 0.01, ***p < 0.001 (NFATc3^+/+^ vs NFATc3^+/-^).

### NFATc3 deficiency mitigates BLM-induced pulmonary inflammation

To determine whether NFATc3 regulates initial stages of BLM-induced pulmonary inflammation, mice were intratracheally treated with BLM and analyzed after 3 and 7 days. As shown in Figure 4A-D, there was a reduction in pulmonary inflammation assessed by the histological changes, decreased immune cell infiltration and protein accumulation in BALF of NFATc3^+/-^ mice compared to NFATc3^+/+^ mice. These results indicate that NFATc3 regulates early stages of BLM-induced pulmonary inflammation.

### NFATc3 expression in pulmonary macrophages promotes BLM-induced fibrosis

Having confirmed the involvement of NFATc3 in lung fibrosis and inflammation, we then determined whether the effect of NFATc3 on the progression of pulmonary fibrosis is mediated through macrophages. To determine the role of macrophage specific NFATc3 on BLM-induced pulmonary fibrosis, we delivered 5 × 10^5^ NFATc3^+/-^ or NFATc3^+/+^ BMDM into the trachea of anesthetized recipient mice (NFATc3^+/+^ or NFATc3^+/-^) that were pre-treated with clodronate liposomes to deplete lung resident macrophage population as described previously (Fig. 5A) [21]. Recipient mice adoptively transferred with NFATc3^+/-^ or NFATc3^+/+^ macrophages were subjected to BLM-induced pulmonary fibrosis and analyzed. Adoptive transfer of NFATc3^+/+^ macrophages to NFATc3^+/+^ and NFATc3^+/+^ groups of mice subjected to BLM-induced IPF showed severely damaged lung tissue (H&E staining). Furthermore, these mice showed a higher number of aggregated fibroblasts and extracellular matrix deposition than the mice that received NFATc3^+/-^ macrophages (NFATc3^+/+^ and NFATc3^+/-^mice) subjected to BLM-induced IPF (Fig. 5B). Consistently, the fibrotic changes in NFATc3^+/+^ recipient mice were further correlated with collagen production assessed by Masson’s trichrome blue staining (Fig. 5C) and fibrosis severity by Ashcroft score (Fig. 5D). As expected, RT-qPCR and immunoblot analysis showed increased expression of the fibrotic markers α-SMA and fibronectin in mice delivered with NFATc3^+/+^ BMDM unlike the mice that received NFATc3^+/-^ macrophages by adoptive transfer (Fig. 5E-H). Similarly, the level of hydroxyproline was significantly increased in mice delivered with NFATc3^+/+^ BMDM unlike the mice that received NFATc3^+/-^ macrophages by adoptive transfer (Fig. 5I). These results demonstrate that macrophage specific NFATc3 plays a pivotal role in regulating BLM-induced pulmonary fibrosis in mice.

**Figure 8.**
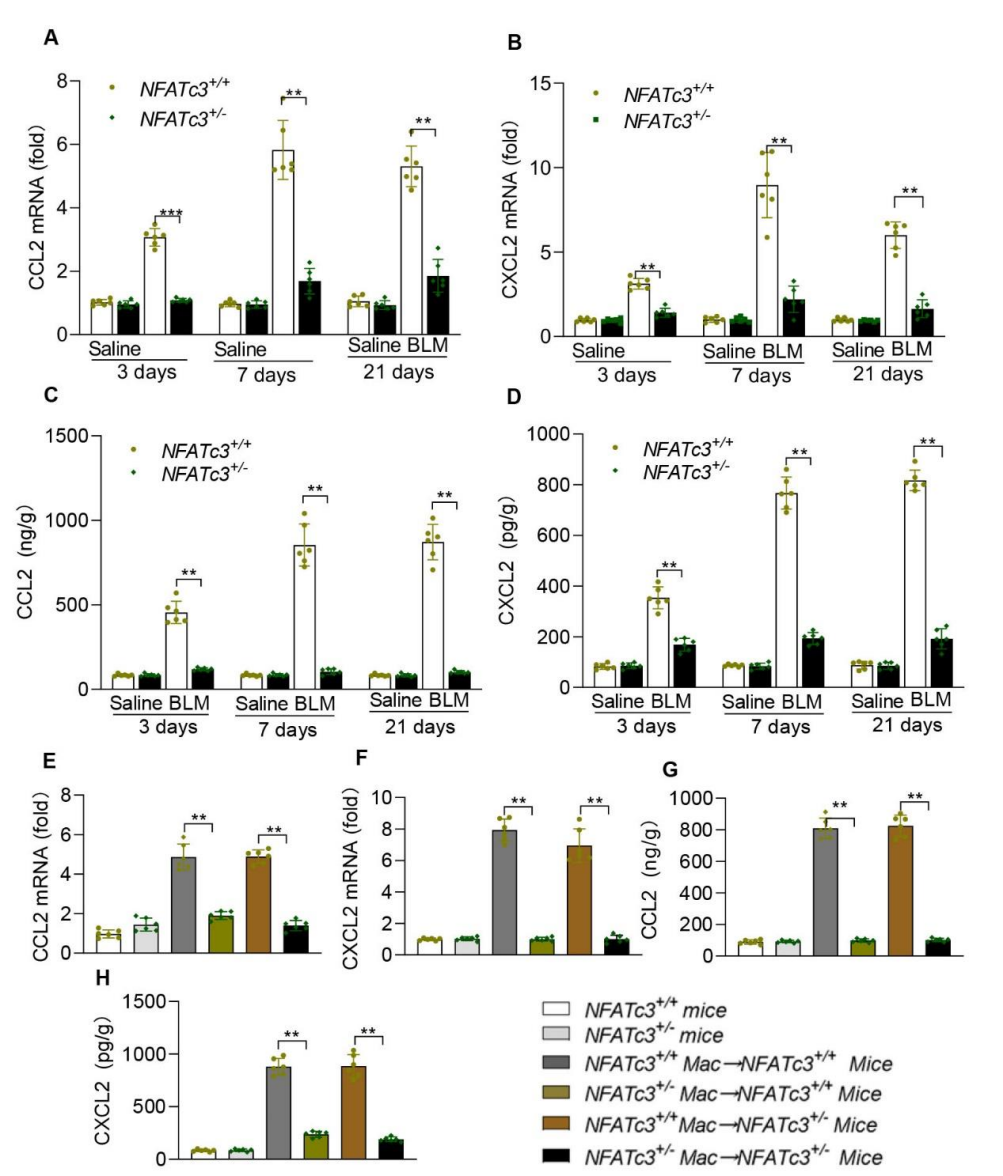
**NFATc3 promoted CCL2 and CXCL2 production in vivo**. (**A, B**) The levels of the cytokines CCL2 and CXCL2 were detected by RT-qPCR in lungs from NFATc3^+/+^ and NFATc3^+/-^ mice that were treated with saline or BLM (i.t) for 3, 7, and 21 days. (**C, D**) The protein levels of CCL2 and CXCL2 were detected by ELISA in lungs from NFATc3^+/+^ and NFATc3^+/-^ mice that were treated with saline or BLM (i.t) for 3, 7, and 21 days. (**E, F**) The levels of the cytokines CCL2 and CXCL2 were detected by RT-qPCR in lungs from recipient mice that were adoptively transferred with NFATc3 deficient (NFATc3^+/-^ to NFATc3^+/+^; NFATc3^+/-^ to NFATc3^+/-^) or NFATc3 sufficient macrophages (NFATc3+/+ to NFATc3^+/-^; NFATc3^+/+^ to NFATc3^+/+^). (**G, H**) The protein levels of CCL2 and CXCL2 were detected by ELISA in the lungs from recipient mice that were adoptively transferred with NFATc3 deficient (NFATc3^+/-^) or sufficient (NFATc3^+/+^) macrophages. Data are shown as mean ± SEM. N=6 for each group, *p < 0.05, **p < 0.01, ***p < 0.001 (NFATc3^+/+^Macrophages→ NFATc3^+/+^/ NFATc3^+/-^ mice vs NFATc3^+/-^Macrophages→ NFATc3^+/+^/ NFATc3^+/-^ mice).

### NFATc3 transcriptionally regulates CCL2 and CXCL2 expression in macrophages and pulmonary fibrosis in mice

The epithelial-macrophage crosstalk plays an important role in pathophysiology of pulmonary inflammation and fibrosis. Alveolar epithelium damaged by BLM or other insults releases a series of cytokines including IL-33 and further activates macrophages to initiate the inflammatory response cascade. To investigate whether IL-33 stimulation or conditioned medium from injured AEC alters macrophage function we have assayed expression of multiple inflammatory and fibrogenic marker genes. As shown in Figure 6A-F and I-N, the expression of inflammatory and fibrogenic cytokines including TNF-α, IL-12p35, IL-12p40, TGF-β and IL-13 were increased but not significantly different between NFATc3^+/+^ and NFATc3^+/-^ macrophages stimulated with AEC conditioned medium or IL-33. Notably, IL-1β was significantly downregulated in response to IL-33 stimulation but remained unaltered in AEC conditioned medium stimulation (Fig. 6B, J). There was a marked increase in expression levels of CCL2 and CXCL2 in IL-33 or AEC conditioned medium stimulated NFATc3^+/+^ macrophages. In contrast, CCL2 and CXCL2 expressions were blunted in the NFATc3^+/-^ compared to the NFATc3^+/+^ macrophages (Fig. 6G, H and Fig. 6O, P). Th2 cytokines are critical factors for generating and maintaining a fibrogenic microenvironment during the development of pulmonary fibrosis. Interestingly, we found that stimulation with the Th2 cytokine mixture IL-4 and IL-13 together upregulated the production of CCL2 and CXCL2 in NFATc3^+/+^ macrophages whereas the same cytokine mix had no impact on CCL2 and CXCL2 expression in NFATc3^+/-^ macrophages (Fig. 6Q, R). These data were further confirmed by measuring CCL2 and CXCL2 protein levels by ELISA in the NFATc3^+/-^ and NFATc3^+/+^ mouse macrophages that were cultured with conditional medium, IL-33 or IL-4 plus IL-13 (Fig. 7A-F).

Then, we assessed whether NFATc3 regulates the production of CCL2 and CXCL2 in mouse *in vivo* pulmonary fibrosis models. As shown in Figure 8A to D, the expression levels of CCL2 and CXCL2 were significantly lower in the lungs of BLM-induced NFATc3^+/-^ fibrosis mice compared to those of NFATc3^+/+^ mice. Furthermore, mice that received NFATc3^+/-^ macrophages by adoptive transfer (NFATc3^+/-^→ NFATc3^+/+^ and NFATc3^+/-^→ NFATc3^+/-^ groups) prior to the treatment with BLM, showed significantly reduced levels of CCL2 and CXCL2 (Fig. 8E-H). Conversely, the expression levels of CCL2 and CXCL2 were markedly augmented in BLM-treated mice that received NFATc3^+/+^ donor macrophages (NFATc3^+/+^→ NFATc3^+/+^, NFATc3^+/+^→ NFATc3^+/-^ groups) (Fig. 8E-H). Taken together, these data support a critical role for NFATc3 in regulating the production of CCL2 and CXCL2 in macrophages during the BLM-induced pulmonary fibrosis progression in mouse models.

### CXCL2 alone can exacerbate BLM-induced pulmonary fibrosis in NFATc3^+/-^ mice

**Figure 9.**
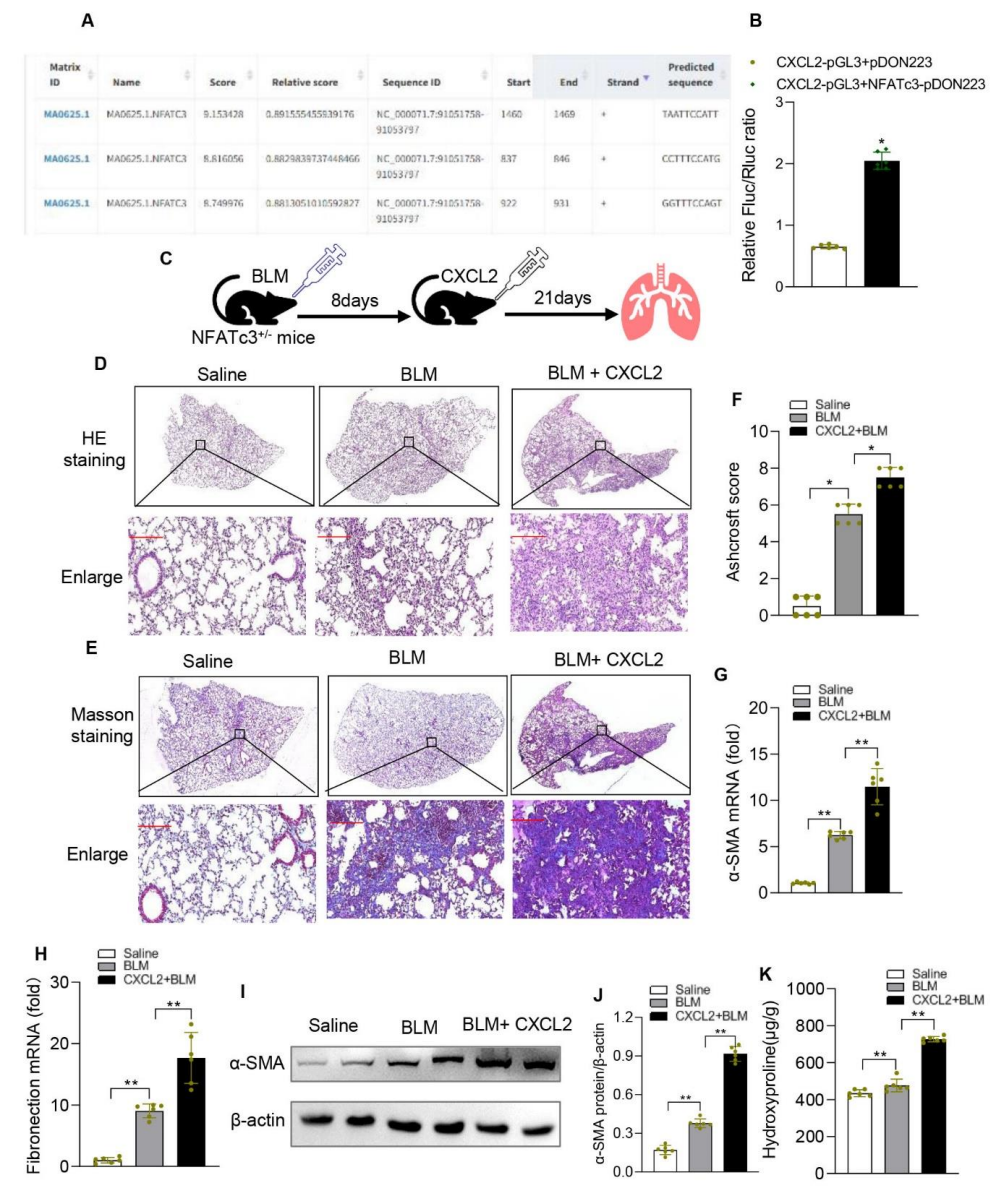
**CXCL2 restores BLM-induced pulmonary fibrosis in NFATc3^+/-^ mice**. (A) Predicted binding sites of NFATc3 in the CXCL2 promoter analyzed using the JASPAR database. (**B**) Quantification of luciferase reporter activity of CXCL2 in Raw264.7 cells transfected with control and NFATc3 plasmids. (**C**) Schematic diagram of the experimental procedure. NFATc3^+/-^ mice were administrated with BLM (i.t) on day 1 and with rmCXCL2 (500ng, i.t) on day 3. Pulmonary fibrosis markers were measured on day 21. (**D**) H&E staining of lung sections (E) Masson trichrome staining to determine collagen deposition (original magnification ×400, scale bar 100μm). (**F**) Comparison of the Ashcroft score among the experimental groups. (**G, H**) The mRNA expression of α-SMA and fibronectin was measured by RT-qPCR. Data are shown as mean ± SEM. (**I, J**) The protein level of ɑ-SMA was analyzed by western blotting quantified using Image J software. (**K**) Hydroxyproline levels in different groups of experimental mice. N=6 for each group, (B) is *p <0.05 (CXCL2-pGL3+NFATc3-pDON223 vs CXCL2-pGL3+pDON223); (F-H, J, K) *p < 0.05, **p < 0.01 (Saline vs BLM and BLM+CXCL2).

To determine the transcriptional regulatory role of NFATc3 in the regulation of CXCL2, the JASPAR database was used to predict the binding sites of NFATc3 in the CXCL2 promoter [22]. Mouse CXCL2 promoter harbored three NFAT consensus binding elements within ~1500 bp region of the promoter (Fig. 9A). Dual-luciferase reporter activity was significantly increased in RAW264.7 cells co-transfected with pGL3-CXCL2 promoter luciferase and NFATc3-pDON223 expression vector, indicating that NFATc3 directly bound the CXCL2 promoter and regulated the transcription of CXCL2 (Fig. 9B). To further confirm the role of CXCL2 in NFATc3-mediated pulmonary fibrosis, we administered recombinant mouse CXCL2 (rmCXCL2), intratracheally into NFATc3^+/-^ mice, 8 days after BLM (i.t) stimulation (Fig. 9C). BLM-induces pulmonary inflammation up to day 7, that transitions into pulmonary fibrosis starting from day 8 [17, 23] . As shown in Figure. 9D to F, pulmonary injury, collagen deposition (Trichrome blue staining) and fibrosis score was further enhanced in BLM-induced NFATc3^+/-^ mice that were administered rmCXCL2. Similarly, rmCXCL2 treatment showed increased expression of α-SMA (Fig. 9G, I, J), fibronectin and hydroxyproline content in BLM-treated NFATc3^+/-^ mice (Fig. 9H, K). These data confirm that NFATc3-mediated CXCL2 production by pulmonary macrophages promotes the progression of pulmonary fibrosis in mouse models.

## DISCUSSION

IPF is a progressive, irreversible lung disease characterized by scarring and thickening of the interstitial tissue in the lung, leading to dyspnea and respiratory failure [2]. Pirfenidone and nintedanib are the only drugs approved recently by FDA for treating IPF [2, 24]. The cellular and molecular mechanisms of IPF pathophysiology are not investigated completely. Therefore, there is a need for identifying cellular mechanisms of IPF to develop targeted therapeutic strategies. In the present study, we demonstrated that IPF patients and mice with the onset of BLM-induced pulmonary fibrosis show increased expression of NFATc3 in lung tissue and macrophages. Notably, NFATc3 was predominantly upregulated and activated in both alveolar and interstitial lung macrophages. NFATc3^+/+^ mice subjected to BLM-induced pulmonary fibrosis showed increased accumulation of fibroblast foci, extracellular matrix protein deposition, fibrotic markers and CCL2 and CXCL2 production. In contrast, all the fibrotic markers, Trichrome blue staining, and Ashcroft score in NFATc3^+/-^ mice subjected to BLM-induced pulmonary fibrosis were significantly attenuated. NFATc3^+/-^ mice that received NFATC3^+/+^ pulmonary macrophages by adoptive transfer or rmCXCL2 alone showed increased pulmonary fibrosis severity and fibrotic gene expression similar to that of WT/ NFATc3^+/+^ mice, suggesting a pivotal role for NFATc3 in the development of lung fibrosis. Our studies show that NFATc3 upregulates CXCL2 transcription by binding directly to its promoter. In summary, these results demonstrate that macrophage specific NFATc3 regulates pulmonary fibrosis development in mouse models.

The bleomycin mouse-IPF model demonstrates that inflammation precedes fibrosis, which occurs through innate and adaptive immunity [25-27]. It is well established in the literature that NFAT proteins regulate both adaptive and innate immunity by fine tuning various cytokine and chemokine genes. Macrophages play a critical role in regulating the lung microenvironment by producing cytokines and chemokines [28, 29]. NFATs modulate inducible gene expression by translocating to nucleus regulated by the Ca^2+^/calmodulin/calcineurin signaling pathway [30, 31]. Previously, we have demonstrated that NFATc3 regulates macrophage effector functions by regulating different cytokines, CCR2, and iNOS during sepsis and acute lung injury [32]. Accumulating evidence supports the role of pulmonary macrophages in the development of pulmonary fibrosis [33-35]. Therefore, we sought to determine the role of NFATc3 in modulating macrophage function in BLM-induced pulmonary fibrosis. It has been shown that activated NFATc2 can promote human lung fibroblast proliferation induced by hypoxia [36]. In contrast, a significant negative correlation was observed between NFATc3 protein expression and lung function in IPF patients [37]. These studies support the fact that NFAT isoforms may play diverse functional roles in different cells.

In the current study, we have observed enhanced expression and activity of NFATc3 in lung tissue of pulmonary fibrosis patients, mouse BLM-induced IPF lungs and pulmonary macrophages. These findings compelled us to investigate whether NFATc3 regulates fibrogenesis through modulating macrophage function. A key finding was that NFATc3 deficiency significantly attenuated BLM-induced pulmonary fibrosis and inflammation, and the adoptive transfer of NFATc3^+/+^ macrophages into NFATc3^+/-^ mice restored cellular and pulmonary fibrotic markers in mouse models of BLM-induced pulmonary fibrosis. It has been established that, damage to epithelial cells leads to the release of inflammatory mediators and initiation of fibrogenesis cascade [38]. Previously, we demonstrated that IL-33 released from epithelial cells induced macrophage activation and contributed to pulmonary fibrosis [17]. In this study, our results show that NFATc3 deficiency substantially reduced the production of chemokines CCL2 and CXCL2 in macrophages under the stimulation of a conditional medium from BLM-treated epithelial cells and rmIL-33 *in vitro*. Given the fact that Th2 cytokines serve as essential regulators for generating and maintaining a fibrotic microenvironment in the late stage of pulmonary fibrosis [39, 40], we stimulated macrophages with cytokines IL-4 and IL-13 together, to stimulate the Th2 microenvironment. IL-4 and IL-13 together increased the expression levels of CCL2 and CXCL2 in NFATc3^+/+^ macrophages unlike NFATc3^+/-^ macrophages. We also show that in vivo expression of these two chemokines was decreased in the lungs of NFATc3^+/-^ mice during both the early inflammatory and late fibrotic phases. Similarly, the adoptive transfer of NFATc3^+/+^ macrophages into NFATc3^+/-^ mice enhanced the CCL2 and CXCL2 levels, which decreased in lungs of NFATc3^+/+^ mice that received NFATc3^+/-^ macrophages. Taken together, these data suggest that the NFATc3 in macrophages is involved in the production of chemokines CCL2 and CXCL2 during IPF pathogenesis. The role of CCL2 in the pathogenesis of pulmonary fibrosis has long been recognized, where elevated levels of CCL2 was observed in the lungs of both IPF patients and animal models [41, 42]. CCL2 may not only exacerbate the early inflammation, but also directly mediate fibrocyte recruitment, thereby contributing to the development of pulmonary fibrosis [42-45]. Therefore, anti-CCL2 gene therapy was found to significantly suppress BLM-induced fibrosis [46]. Chemokine (CC motif) receptor-2 (CCR2), was also reported to be important for the IPF progression. CCR2 deficient mice displayed decreased pulmonary fibrosis due to decreased infiltration of macrophages and expression of different macrophage specific metalloproteases during bleomycin and fluorescein isothiocyanate exposure [47]. Previously we have demonstrated that NFATc3 can directly bind to the promoter of CCR2 in macrophages upon LPS stimulation [48]. Few studies have addressed the mechanism of CXCL2 in the lung during the development of pulmonary fibrosis. An early study reported that CXCL2 regulates angiogenesis and neutralizing anti-CXCL2 antibody significantly reduced BLM-induced pulmonary fibrosis [49]. However, this study does not report the molecular mechanism of CXCL2 activation. Our results demonstrate that NFATc3 transcriptionally regulates CXCL2 by directly binding to its promoter DNA. In addition, rmCXCL2 delivered through i.t instillation increased fibrogenesis in lung tissue of NFATc3^+/-^ mice.

In conclusion, we demonstrated that upregulated NFATc3 expression is a characteristic manifestation during the course of pulmonary fibrosis. Therefore, mice deficient in NFATc3 are protected from BLM-induced lung inflammation and fibrosis. Mechanistic studies revealed that NFATc3 is involved in the pathogenesis of pulmonary fibrosis by promoting CCL2 and CXCL2 production in response to damaged epithelial cells, IL-33 and Th2 cytokine stimulation. Our data support targeting NFATc3 as a novel strategy for prevention and treatment of pulmonary fibrosis in clinical settings.
